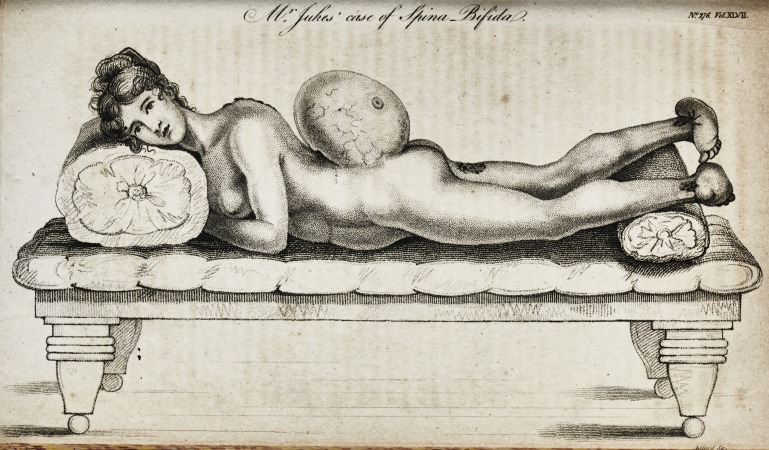# Description of a Singular Case of Spina Bifida, in a Female, Nearly Twenty Years of Age, Now Living in the Horseferry-Road, Westminster

**Published:** 1822-02

**Authors:** E. Jukes

**Affiliations:** Surgeon. With a Drawing of the Disease.


### PATHOLOGY.

ART. III.—*Description of a singular Case of Spina Bifida, in a Female, nearly Twenty Years of Age, now living in the Horseferry-Road, Westminster.* By E. JUKES, Surgeon. With a Drawing of the Disease.

ANN SELBY, aged about nineteen years, residing with her parents in the Horseferry-Road, Westminster, was born with a slight membranous tumor, the size of a small pigeon's egg, situated on the superior portion of the sacrum, and below the third lumbar vertebra. She was, in every other respect, well formed, and continued to grow in the most satisfactory manner, until the age of eight years; walking about the house, without much inconvenience from the tumour, the latter being supported by an appropriate bandage. At this period, the right foot began to contract, and turn inwards and upwards, as represented in the plate; the tumour continuing, at the same time, to increase.

At eleven years of age, menstruation became fully established in the usual manner.

At fourteen, the menses ceased to flow from the natural passage, and made their appearance through certain deep ulcerations, which had formed, and still exist, in the ankles and the fleshy part of the left thigh, as marked in the plate, letter A. This fact, respecting menstruation, is incontestible, and has been verified by several practitioners.

When fifteen years old, the left foot of the patient contracted also, and turned towards the right; as shewn in the drawing.

The tumor has now reached the size delineated in the accompanying engraving, proportionate to the other parts of the figure. Its dimensions were taken a few days back.

The present appearance of the tumor is well represented in the plate; and a cast of it, which I took at the same time, is deposited in the museum of St. Thomas's Hospital.

---

* The vertical line of the tumor measures thirty inches; the greatest circumference, twenty-eight inches; and the lesser, twenty inches. Assuming Mr. Juke's dimensions as the basis of a calculation for ascertaining the quantity of the fluid contents of the tumor, we have 226.836 cubic inches = 14 + lb. troy = 11.37 lb. avoirdupois, or one gallon, three pints, and five ounces.—A. B. G.

Mr. Jukes' case of Spina Bifida.

Adlard Sc.

The tumor is transparent, equally distended, fluctuating, and occasionally larger than at other times. Figure B is intended to represent a kind of horny scale, not quite as large as half-a-crown, which occasionally peels off from the surface of the tumor, and, leaving the skin without its cuticle, gives rise to a very perceptible oozing of a serous fluid, which, after a short time, ceases; some of it drying in thin layers, and leaving the horny scale above described.

Neither the urine nor the feces have ever been retained, but pass involuntarily, and almost constantly.*

During the last two years, the health of the patient has been sensibly declining, and, from being corpulent, she is now wasted almost to a skeleton. The stomach rejects every kind of food, unless accompanied by a large quantity of gin, which forms her only drink, and, indeed, almost her only support. The development of her intellectual faculties seems in no way to have been influenced by the formidable disease with which she is afflicted, and which she bears with becoming resignation.†

*Great Peter-street, Westminster; January* 1822.

* The paralytic state of the lower intestines and bladder, in cases of spina bifida, has been remarked by almost every author who has had an opportunity of observing that disease. In our own practice, we have had occasion to see five cases of this malady, one of which, very recently, at the Westminster General Dispensary; and in all of them defecation and micturition took place involuntarily. In another case, which we carefully dissected in the presence of our colleagues, Dr. Macleod and Mr. A. C. Hutchison, all traces of spinal nerves were lost below the tumor; on the inner surface of which, the terminations of those nerves were beautifully arranged and implanted. The preparation is now in our collection at that Institution.—A. B. G.

† In a very recent volume of the Transactions of the Imperial Academy of Sciences of St. Petersburgh, which we had occasion to see in the library of the Royal Society, there is a very interesting memoir on spina bifida, with very remarkable cases of that disease, by a Russian physician; which we shall take some future opportunity of noticing at length in our Journal.—A. B. G.

## Figures and Tables

**Figure f1:**